# 1,2,3,4,6-Penta-O-galloyl-d-glucose Interrupts the Early Adipocyte Lifecycle and Attenuates Adiposity and Hepatic Steatosis in Mice with Diet-Induced Obesity

**DOI:** 10.3390/ijms23074052

**Published:** 2022-04-06

**Authors:** Ashish Rao Sathyanarayana, Chung-Kuang Lu, Chih-Chuang Liaw, Chia-Chuan Chang, Hsin-Ying Han, Brian D. Green, Wei-Jan Huang, Cheng Huang, Wen-Di He, Lin-Chien Lee, Hui-Kang Liu

**Affiliations:** 1 Ph.D. Program for the Clinical Drug Discovery from Herbal Medicine, College of Pharmacy, Taipei Medical University, Taipei 110, Taiwan; ashrao110.ar@gmail.com (A.R.S.); wjhuang@tmu.edu.tw (W.-J.H.); 2 Division of Chinese Medicinal Chemistry, National Research Institute of Chinese Medicine, Ministry of Health and Welfare, Taipei 112, Taiwan; cklu@nricm.edu.tw; 3 Department of Marine Biotechnology and Resources, National Sun Yat-sen University, Kaohsiung 804, Taiwan; ccliaw@mail.nsysu.edu.tw (C.-C.L.); wendy880323@gmail.com (W.-D.H.); 4 Department of Pharmacy, National Taiwan University, Taipei 10617, Taiwan; chiachang@ntu.edu.tw; 5 Department of Bioscience and Biotechnology, National Taiwan Ocean University, Keelung 202, Taiwan; hsying619@gmail.com; 6 Institute for Global Food Security, School of Biological Sciences, Queen’s University Belfast, Belfast BT7 1NN, UK; b.green@qub.ac.uk; 7 Department of Biotechnology and Laboratory Science in Medicine, National Yang-Ming University, Taipei 112, Taiwan; chengh@ym.edu.tw; 8 Department of Earth and Life Sciences, University of Taipei, Taipei 11036, Taiwan; 9 Department of Physical Medicine and Rehabilitation, Cheng Hsin General Hospital, 45, Cheng Hsin Street, Taipei 112, Taiwan; 10 Traditional Herbal Medicine Research Center, Taipei Medical University Hospital, Taipei 110, Taiwan; 11 Division of Basic Chinese Medicine, National Research Institute of Chinese Medicine, Ministry of Health and Welfare, 155-1 Li-Nong Street, Section 2, Taipei 112, Taiwan

**Keywords:** 1,2,3,4,6-penta-O-galloyl-d-glucose, obesity, adipocyte lifecycle, apoptosis, hepatic steatohepatitis

## Abstract

Phytochemicals that interrupt adipocyte lifecycle can provide anti-obesity effects. 1,2,3,4,6-penta-O-galloyl-d-glucose (PGG) is a tannin with two isomers that occurs widely in plants and exhibits various pharmacological activities. The aim of the investigation is to comprehensively examine effects of PGG isomer(s) on adipocyte lifecycle and diet-induced obesity. Human mesenchymal stem cells (hMSC), 3T3-L1 fibroblasts, and H4IIE hepatoma cells were used to determine the effects of PGG isomers on cell viability and adipogenesis. Mice with diet-induced obesity were generated from male C57/BL6 mice fed with a 45% high fat diet. Oral administration of β-PGG (0.1 and 5 mg/kg) lasted for 14 weeks. Viability was reduced by repeated PGG treatment in hMSC, preadipocytes, and cells under differentiation. PGG mainly induces apoptosis, and this effect is independent of its insulin mimetic action. In vivo, administration of β-PGG attenuated shortening of the colon, hyperlipidaemia, fat cells and islet hypertrophy in DIO mice. Hepatic steatosis and related gene expression were improved along with glucose intolerance. Increased serum adiponectin, leptin, and glucagon-like peptide-1 levels were also observed. In conclusion, repeated PGG treatment interrupts the adipocyte lifecycle. PGG administration reduces adiposity and fatty liver development in DIO mice, and therefore, PGG could aid in clinical management of obesity.

## 1. Introduction

Obesity is a form of malnutrition and is one of three pandemics threatening global health [1]. In 2016, 1.9 billion people were overweight, and 650 million people were obese. It is particularly concerning that more than 340 million children and adolescents are either overweight or obese [2]. Overconsumption of fatty and sugary food and reduced physical activity are the key drivers of obesity. The development of obesity leads to abnormal morphology and metabolic function of adipocytes, and to the dysregulation of plasma non-esterified fatty acids and cytokines. In many cases, this leads to other co-morbidities such as hypertension, atherosclerosis, diabetes, and some cancers [3,4]. Conventional treatment involves changing lifestyle (to improve diet and exercise), but also the use of anti-obesity drugs and various surgical interventions to improve disease prognosis; however, such approaches are neither economical nor effective [5].

The adipocyte lifecycle hypothesis proposes that the metabolic consequences of obesity are dependent on whether adipose tissue enlargement occurs mainly by an increase in adipocyte size [6,7]. This in turn affects plasma fatty acid homeostasis and the number of adipocytes to be differentiated from pre-adipocytes [6,7]. Targeting the adipocyte lifecycle prevents the initiation and the progression of obesity and related diseases [8]. Dietary phytochemicals, such as genistein, conjugated linoleic acid, epigallocatechin gallate, quercetin, and resveratrol have been shown to affect different stages of the adipocyte lifecycle by promoting apoptosis of pre-adipocytes and adipocytes, by inhibiting adipogenesis in pre-adipocytes, and by stimulating lipolysis in mature adipocytes. A combination of new drugs based on similar chemical scaffolds to the aforementioned compounds may be effective in preventing or treating obesity.

1,2,3,4,6-penta-O-galloyl-d-glucose (PGG) is a hydrolysable tannin precursor with two isomers (α and β). β-PGG is the dominant form. It occurs widely in plants and has various pharmacological activities, including anti-oxidant, anti-cancer, anti-viral, anti-inflammation and anti-diabetic activity [9]. PGG is also present in herbs that are used in complementary and alternative medicine. Tannic acids consisting of PGG from *Lagerstroemia speciosa* (banaba) could inhibit adipocyte differentiation [10]. Li Y and colleagues reported that PGG binds to insulin receptors to promote glucose uptake in 3T3-L1 adipocytes and improves glucose tolerance in diabetic and obese mice [11]. A previous study by Yuan et. al. showed that PGG is an insulin mimetic in Paeoniae Rubra Radix that results in suppressing hepatic phosphoenolpyruvate carboxykinase gene transcription in cell study [12]. PGG containing herbal extract is also potent against hyperglycaemia, hyperlipidaemia, obesity and fatty liver in mice with type 2 diabetes [13].

In terms of the physiological effects of insulin on adipocytes, insulin promotes adipogenesis, stimulates glucose uptake, and inhibits hormone-sensitive lipase [14,15,16]. The insulin mimetic action of PGG may potentially counter the probable anti-obesity effect of PGG. This study investigated the relationship between PGG’s insulin mimetic action and its anti-obesity effect by determining the biological activity of PGG at each stage of the adipocyte lifecycle. It also examined the influence of insulin action on PGG’s ability to intervene in the adipocyte lifecycle. Finally, the overall biological effect of PGG on mice with diet-induced obesity (DIO) was evaluated comprehensively.

## 2. Results

### 2.1. PGG Effects on the Adipocyte Lifecycle

**Figure 1:**
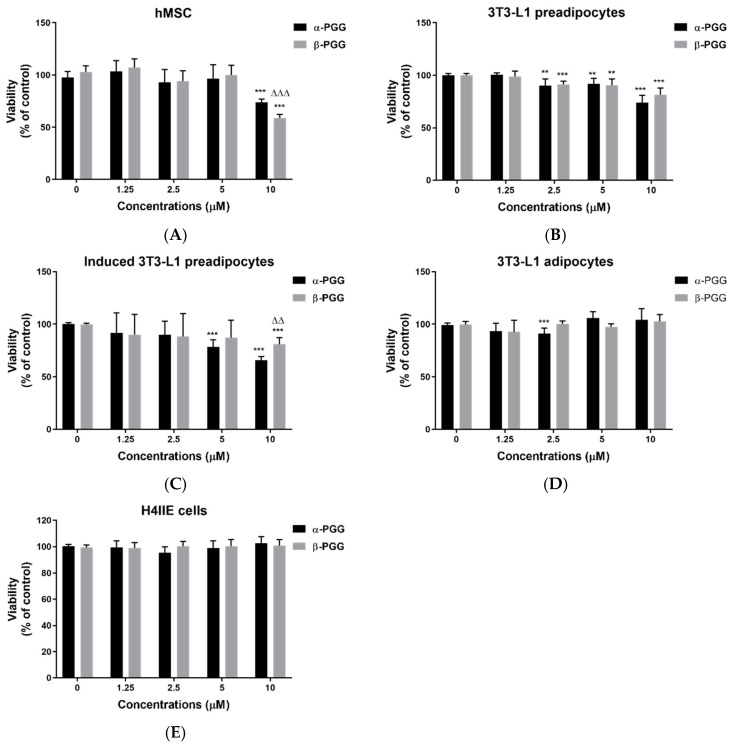
1,2,3,4,6-Pentagalloylglucose (PGG) treatment reduced cell viability in various stages of the adipocyte lifecycle. Effects of α-or β-PGG on cell viability of (**A**) human mesenchymal stem cells (hMSCs; *n* = 5), (**B**) 3T3-L1 fibroblasts/pre-adipocyte (*n* = 5), (**C**) differentiating 3T3-L1 cells (*n* = 14), (**D**) differentiated 3T3-L1 adipocytes (*n* = 9), and (**E**) rat hepatoma H4IIE cells (*n* = 5). Data are shown as mean ± SEM. ** *p* < 0.01 and *** *p* < 0.001 compared to control (vehicle). ^∆∆^ *p* < 0.01 and ^∆∆∆^ *p* < 0.001 compared with corresponding α-PGG group.

The biological activity of repeated α-PGG or β-PGG treatment in various stages of the adipocyte lifecycle was compared. Confirmation of the chemical structure and purity of α-PGG and β-PGG was made before carrying out the experiments (Figure S1). PGG treatment was administrated three times, once every two days, prior to the cell assay. The PGG administration scheme was repeated for all stages of the adipocyte lifecycle. Figure 1A shows that both α- and β-PGG reduce the viability of hMSC (*p* < 0.001). β-PGG has a greater effect than α-PGG (*p* < 0.001). When 3T3-L1 fibroblasts/pre-adipocytes were subjected to PGG treatment, the viability of 3T3-L1 cells was reduced as the concentrations increased from 2.5 to 10 μM. The effect observed was the same for both isomers (Figure 1B). When PGG treatment was performed during the adipogenesis stage, there was a decrease in viability at 5 μM of α-PGG (*p* < 0.001) and at 10 μM of β-PGG (*p* < 0.001; Figure 1C). For differentiated adipocytes, the viability was mostly unaffected by PGGs (Figure 1D). The viability of hepatoma H4IIE cells was also unaffected by PGG treatment (Figure 1E).

**Figure 2:**
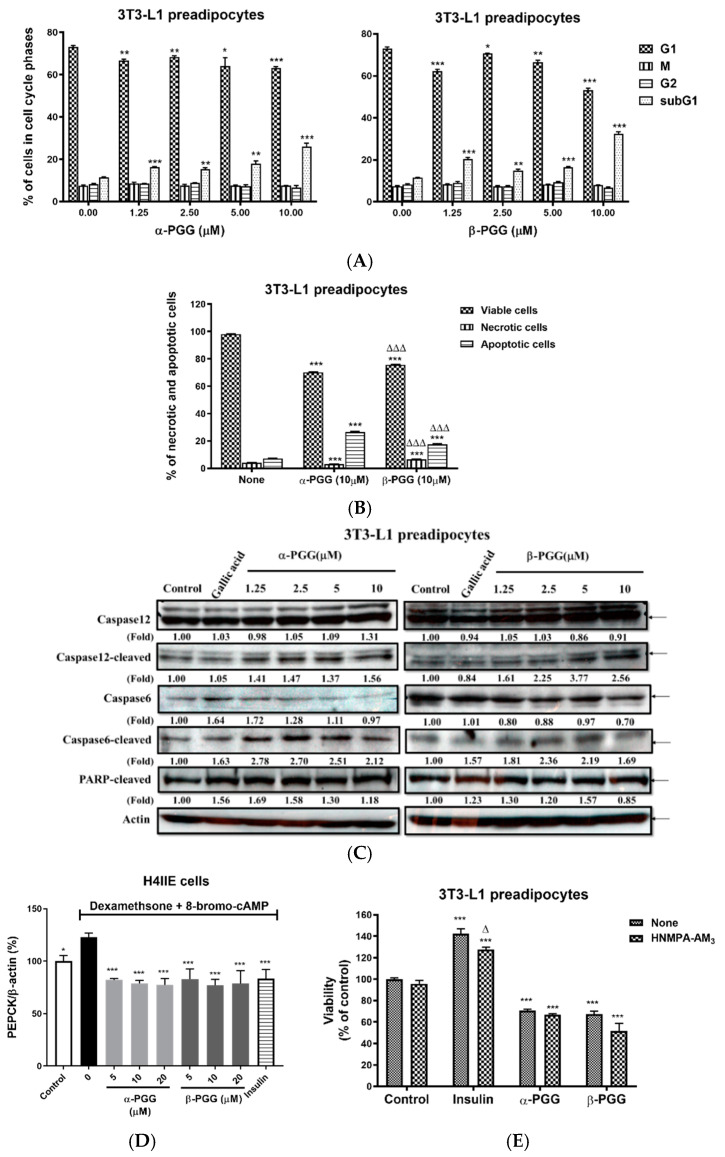
The induction of pre-adipocyte cell death by PGG is independent from its insulin mimetic action. 3T3-L1 pre-adipocyte was treated with α- and β-PGG and evaluated for (**A**) cell cycle analysis, (**B**) annexin-V (AV) and propidium iodide (PI) staining (*n* = 3), and (**C**) apoptosis-related protein biomarkers. The biological activity of PGG to suppress (**D**) phosphoenopyruvate carboxykinase (PEPCK; *n* = 3) mRNA expression and to reduce (**E**) cell viability with or without HNMPA-AM_3_ (*n* = 4) were also measured. Data are shown as mean ± SEM.* *p* < 0.05, ** *p* < 0.01, and *** *p* < 0.001 compared to control (vehicle). ^∆^ *p* < 0.05 and ^∆∆∆^ *p* < 0.001 compared with corresponding α-PGG group or non-HNMPA-AM_3_ condition.

The characteristics of PGG-induced cell death were determined. Figure 2A shows a cell cycle analysis of 3T3-L1 preadipocytes that are treated with PGG. Both α- and β-PGG significantly reduce the cell population in the G1 phase and increase the cell population in the subG1 phase. When annexin-V (for apoptotic cells) and propidium iodide (for necrotic cells) are used for staining, the apoptotic and necrotic cell populations increase after PGG treatment. The apoptotic cell population was greater than the necrotic population. For PGG isomers, α-PGG increased the apoptotic cell population and decreased the necrotic population compared to β-PGG (Figure 2B). Figure 2C shows that both PGG isomers increased the cleaved forms of caspase 12, caspase 6 and Poly ADP-ribose polymerase (PARP) proteins. In contrast, gallic acid treatment only increased cleaved caspase 6 and PARP protein. Figure 2D shows that dexamethasone and 8-bromo-cAMP-induced hepatic phosphoenolpyruvate carboxykinase (PEPCK) mRNA expression was inhibited by insulin (10 nM) and both PGG isomers. However, insulin treatment increased the viability of 3T3-L1 preadipocytes, and this effect was dependent on the insulin receptor inhibitor (HNMPA-AM_3_). The reduction in the viability by α- or β-PGG treatment is unaffected by HNMPA-AM_3_ (Figure 2E).

### 2.2. Effects of PGG on Adipogenesis

**Figure 3:**
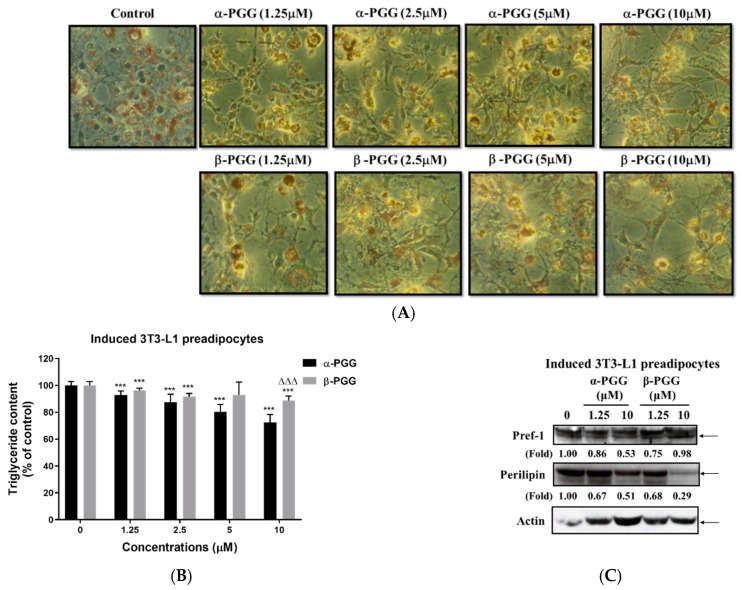

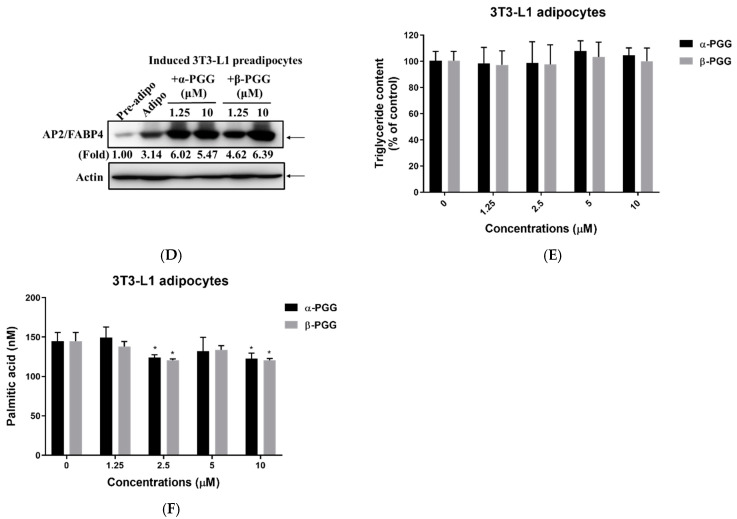
Effect of PGG on the adipogenesis and lipid content of 3T3-L1 cells. During adipogenesis, 3T3-L1 cells were treated with α-or β-PGG. (**A**) Morphology with oil-red O staining, (**B**) quantification of triglyceride content (*n* = 6) and (**C**) Pref-1 and perilipin protein expression, as measured at the end of the differentiation process. (**D**) aP2/FABP4 protein expression in pre-adipocytes and during adipogenesis in the presence of PGGs was compared. By using differentiated 3T3-L1 adipocytes, the effect of PGG on (**E**) triglyceride content (*n* = 6) and (**F**) free fatty acid levels (*n* = 3) were evaluated. Data are shown as mean ± SEM. * *p* < 0.05 and *** *p* < 0.001 compared to control (vehicle). ^∆∆∆^ *p* < 0.001 compared with corresponding α-PGG group.

Figure 3A,B shows the inhibitory effect of PGG treatment on the TG content of the 3T3-L1 cells after the induction of adipogenesis. The morphology of differentiated 3T3-L1 adipocytes stained with oil-red O shows that fully differentiated 3T3-L1 adipocytes with multiple oil-droplets accumulate within the cytoplasm. In addition, adipocytes that are treated with PGG are poorly differentiated, and the morphology has a fibroblast-like shape. In terms of the triglyceride content of these cells, treatment with α- and β-PGG resulted in a significant decrease in TG content at concentrations between 1.25 and 10 μM. α-PGG had a greater effect than β-PGG at 10 μM. Figure 3C shows that at the end of the PGG treatment, pref-1 protein expression is decreased in cells that are treated with α-PGG (10 μM) and β-PGG (1.25 μM). However, perilipin protein expression was also decreased after α- or β-PGG treatment at 10 μM. Once 3T3-L1 preadipocytes transform into differentiated 3T3-L1 adipocytes, adipocyte biomarker adipocyte Protein 2 (aP2), also known as adipocyte lipid–binding protein (FABP4), was expressed in differentiated 3T3-L1 adipocytes. In the presence of α-PGG and β-PGG, aP2 expression was not reduced but was significantly increased after PGGs treatment (Figure 3D). When PGG treatment was employed for differentiated 3T3L-1 adipocytes, the triglyceride content of differentiated 3T3-L1 adipocytes was unaffected by PGG treatment (Figure 3E). However, significantly less free fatty acid was released from differentiated 3T3L-1 adipocytes after α- or β-PGG treatment between concentrations of 2.5 and 10 μM (*p* < 0.05; Figure 3F).

### 2.3. Metabolic Effects of PGG In Vivo in DIO Mice

**Figure 4:**
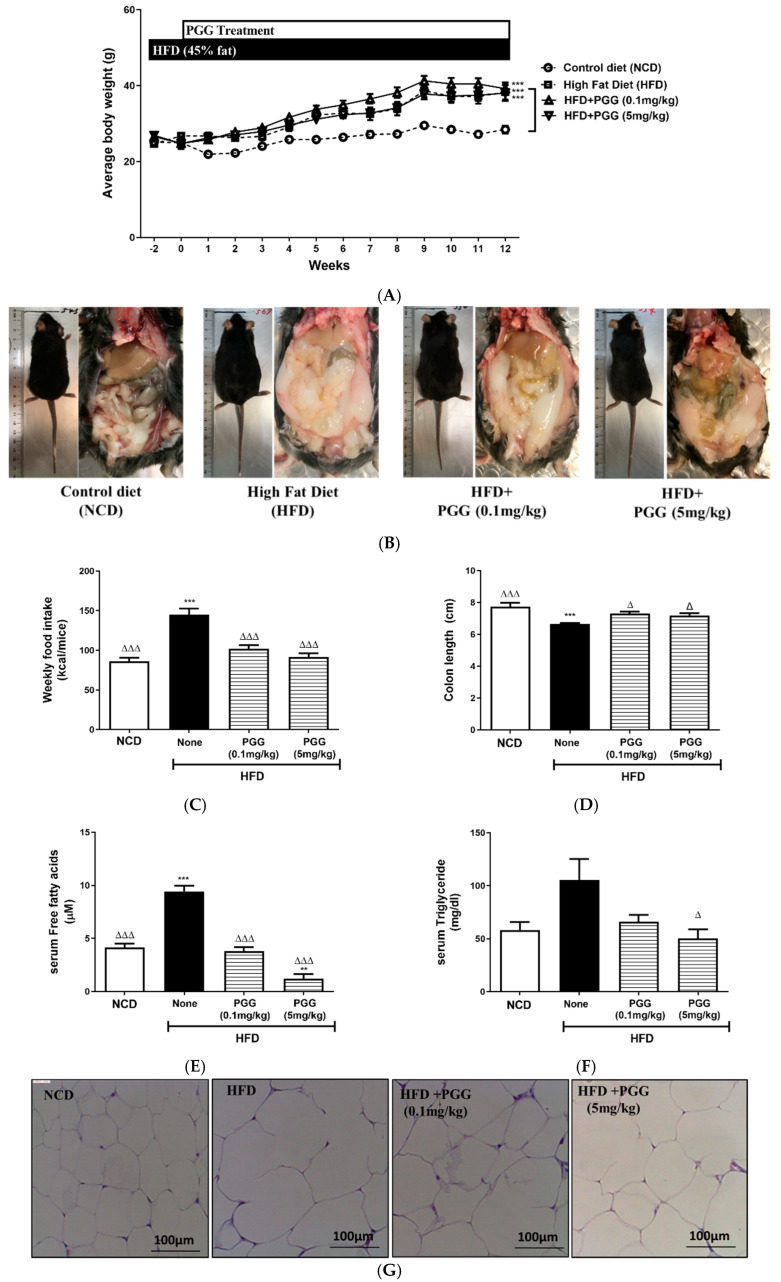

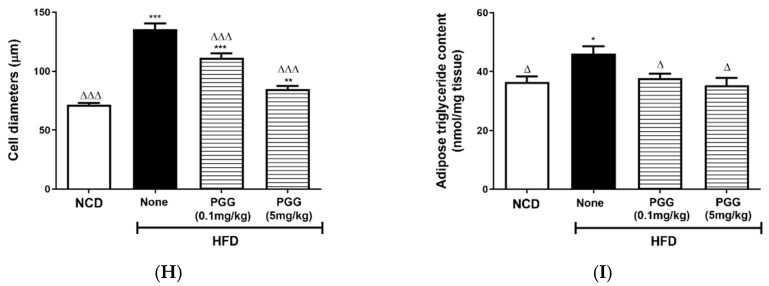
Effect of 12 weeks of β-PGG supplementation on body weight, serum lipids and adipose tissue in DIO mice. (**A**) The weekly body weight of DIO mice treated with PGG (*n* = 6), (**B**) images of dissected mice at the end of the PGG treatment, (**C**) average weekly food intake (*n* = 6), (**D**) colon length, (**E**) serum free fatty acids, (**F**) serum triglyceride, (**G**) images of haematoxylin–eosin-stained adipose tissue, (**H**) diameter of adipocyte cells (*n* > 50 per group), and (**I**) adipose tissue TG content. Data are shown as mean ± SEM (*n* = 4). * *p* < 0.005, ** *p* < 0.01, and *** *p* < 0.001 compared with control diet (NCD) group. ^∆^ *p* < 0.05 group and ^∆∆∆^ *p* < 0.001 compared with HFD (none) group.

In terms of the biological effects of β-PGG supplementation on diet-induced obese (DIO) mice, dietary manipulation by changing the normal chow diet to a high-fat diet (45% energy from fat) was initiated two weeks before PGG supplementation. β-PGG that was isolated from the roots of *Paeonia lactiflora* Pall was used for animal studies. PGG oral administration was then continued for 12 weeks. Figure 4A shows that weekly body weight increased significantly for the high-fat diet group (HFD) after 12-week treatment with or without PGG, compared to the controlled diet (NCD) group. The outlook of mice and abdominal fat pad accumulation show that there was a dose-dependent attenuation of fat pad accumulation (Figure 4B).

The increased weekly food intake (Figure 4C), shortening of the colon (Figure 4D), elevated serum free fatty acid (Figure 4E), and elevated serum triglyceride (Figure 4F) for DIO mice are all attenuated after PGG treatment. Figure 4G,H shows the histology of adipose tissue and shows that hypertrophy of fat cells in DIO mice is ameliorated by PGG treatment in a dose-dependent manner. Therefore, triglyceride content of adipose tissue in DIO mice that are treated with PGG is significantly decreased (Figure 4I).

### 2.4. Effects of PGG on Hepatic Steatosis and Islet Function on DIO Mice

**Figure 5:**
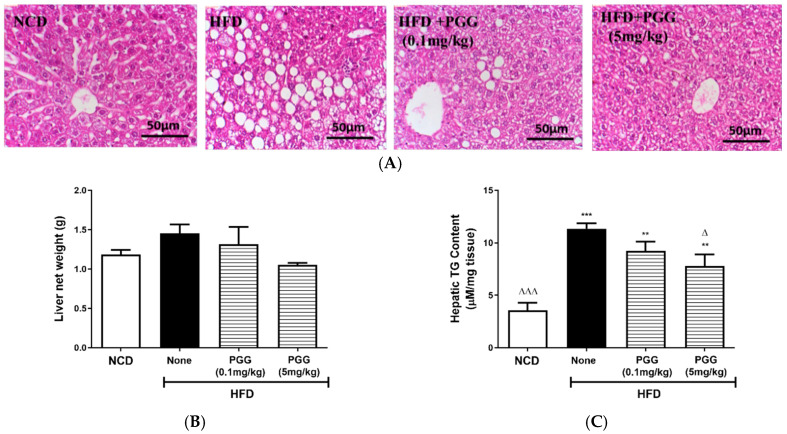

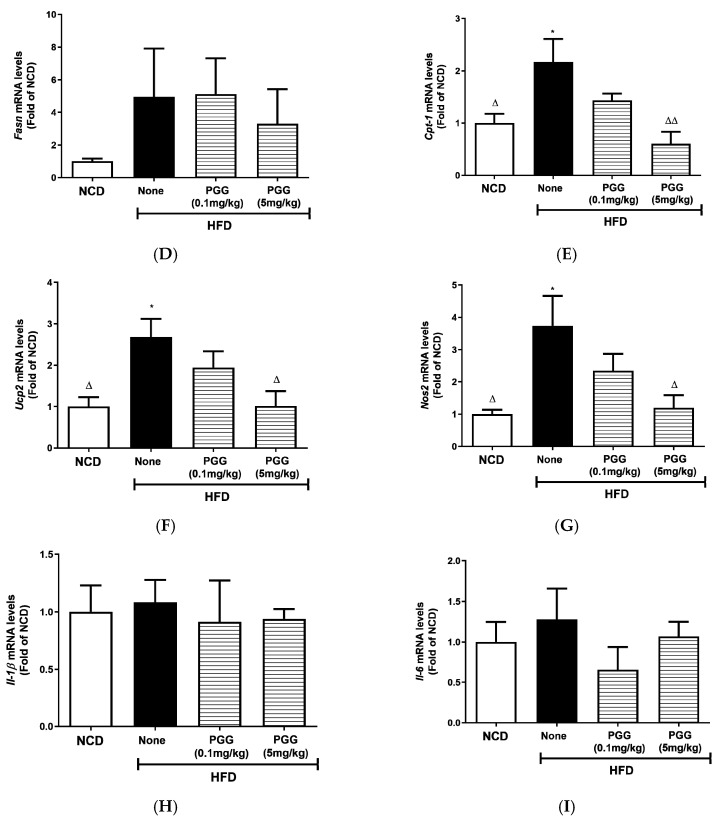
Effect of 12 weeks of β-PGG supplementation on diet-induced hepatic steatosis and gene expression in DIO mice. (**A**) Images of haematoxylin–eosin-stained liver tissue, (**B**) liver weight, (**C**) hepatic liver TG content and gene expression of (**D**) fatty acid synthase (*Fasn*), (**E**) carnitine palmitoyltransferase 1 (*Cpt-1*), (**F**) mitochondrial uncoupling protein 2 (*Ucp-2*), (**G**) nitric Oxide Synthase 2 (*Nos2*), (**H**) interleukin 1 beta (*Il-1β*), and (**I**) interleukin 6 (*Il-6*). Data are shown as mean ± SEM. (*n* = 4) * *p* < 0.05, ** *p* < 0.01 and *** *p* < 0.001 compared with control diet (NCD) group. ^∆^ *p* < 0.05 group, ^∆∆^ *p* < 0.01, and ^∆∆∆^ *p* < 0.001 compared with HFD (none) group.

In terms of the effects of PGG treatment on hepatic steatosis on DIO mice, Figure 4A shows that body weight from DIO mice is significantly increased regardless of PGG treatment (*p* < 0.001). The histological appearance of lipid accumulation in the liver of DIO mice is attenuated by PGG treatment in a dose-dependent manner (Figure 5A). Although liver weight among each group had no statistical difference (Figure 5B), there was decreased hepatic TG content in DIO mice that were treated with PGG (5 mg/kg; Figure 5C). Measurement of gene expression that was related to lipid metabolism and inflammation shows that elevated hepatic CPT-1 (Figure 5E), UCP-2 (Figure 5F) and NOS2 (Figure 5G) mRNA expression in DIO mice was significantly reduced after PGG (5 mg/kg) treatment, but there was no difference between groups in the expression of hepatic FASN (Figure 5D), IL-1β (Figure 5H), and IL-6 (Figure 5I).

**Figure 6:**
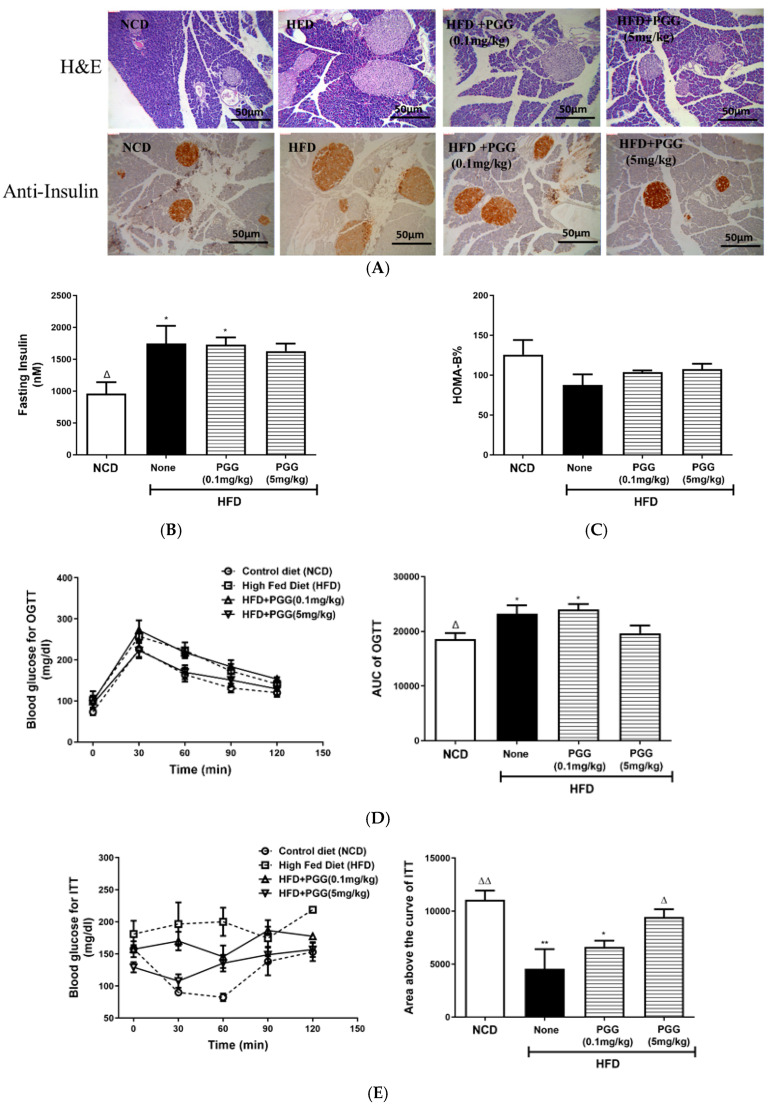

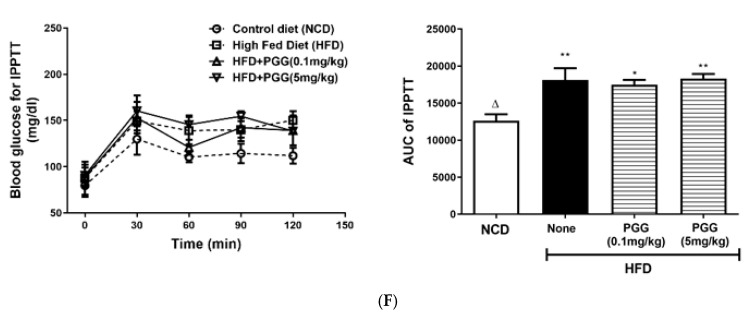
Effect of 12 weeks of β-PGG supplementation on pancreatic islet function and glycaemic control in DIO mice. (**A**) Images of haematoxylin–eosin- (upper) and insulin- (lower) stained pancreatic tissues, (**B**) fasting insulin, (**C**) HOMA-B%, (**D**) glucose tolerance test (OGTT; left) and corresponding area under curve (AUC; right), (**E**) insulin tolerance test (ITT; left) and corresponding area above curve (right), and (**F**) pyruvate tolerance test (IPPTT; left) and corresponding area under curve (AUC; right). Data are shown as mean ± SEM (*n* = 4). * *p* < 0.05 and ** *p* < 0.01 compared with control diet (NCD) group. ^∆^ *p* < 0.05 group and ^∆∆^ *p* < 0.01 compared with HFD (none) group.

In terms of the effect of PGG supplement on the pancreatic islet function and glycaemic control in DIO mice, hypertrophic islet morphology in DIO mice was ameliorated by PGG treatment (Figure 6A upper panel). However, insulin content of islets was increased in DIO mice treated with PGG (5 mg/kg; Figure 6A, lower panel). Fasting insulin (Figure 6B) and beta-cell function index HOMA-B% (Figure 6C) in DIO mice was similar to that of DIO mice that were treated with PGG, but there was an improvement in both glucose tolerance (no difference when compared to NCD; Figure 6D) and insulin tolerance (*p* < 0.05 when compared to HFD (none); Figure 6E) in DIO mice that were treated with PGG (5 mg/kg). Finally, PGG treatment did not improve pyruvate intolerance in DIO mice, as shown in Figure 6F.

### 2.5. Effects of PGG on Endocrine Hormone Levels in DIO Mice

**Figure 7:**
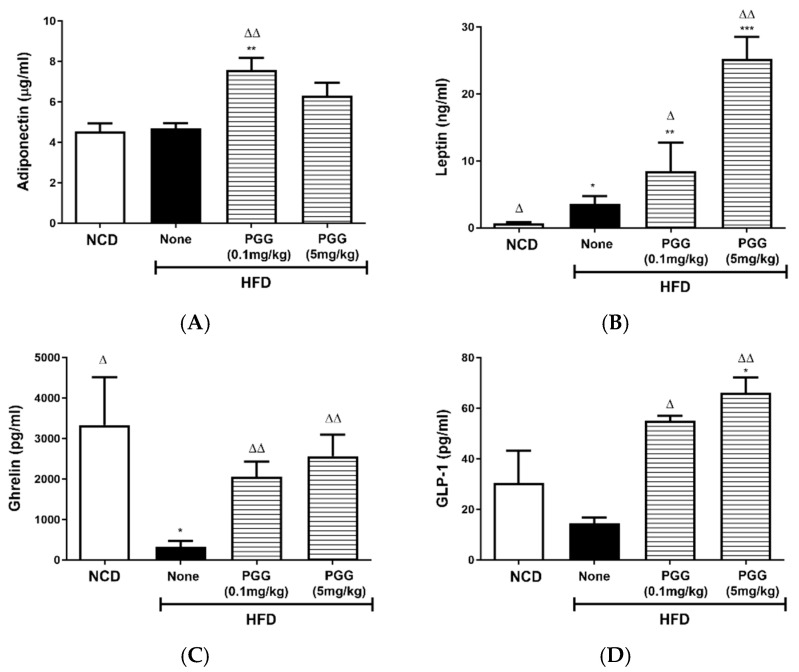

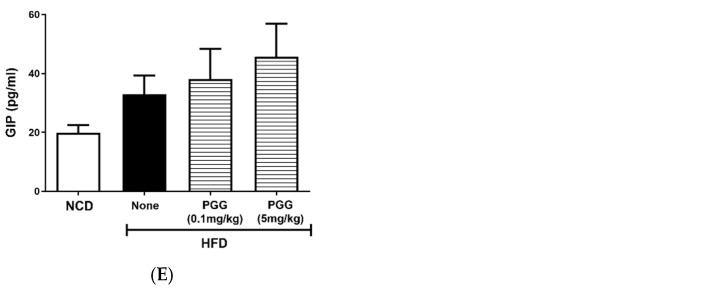
Effect of 12 weeks of β-PGG supplementation on endocrine hormones in DIO mice. Serum levels for (**A**) adiponectin, (**B**) leptin, (**C**) ghrelin, (**D**) glucagon-like peptide-1 (GLP-1), and (**E**) gastric inhibitory peptide (GIP) were measured. Data are shown as mean ± SEM (*n* = 4). * *p* < 0.05, ** *p* < 0.01 and *** *p* < 0.001 compared with control diet (NCD) group or vehicle control group. ^∆^ *p* < 0.05 group and ^∆∆^ *p* < 0.01 compared with HFD (none) group.

Serum adiponectin levels were significantly elevated (*p* < 0.01) in DIO mice that were treated with PGG (0.1 m/kg; Figure 7A). Compared to the HFD group, the presence of PGG resulted in a significant elevation in serum leptin, ghrelin and GLP-1 levels (Figure 7B–D). GIP levels for all groups were not statistically different (Figure 7E).

## 3. Discussion

Two anomeric forms of PGG occur in nature, but β-PGG is the dominant form, and it occurs in a wider variety of plants than α-PGG [17]. Our study was able to compare the two isomers with similar purity in vitro, but for in vivo studies, it was only possible to obtain sufficient quantities of β-PGG. This is not immediately problematic given that this is the isomer that humans most commonly consume. We have demonstrated that both isomers affect the viability of hMSC, preadipocytes, and differentiating preadipocytes. There is a difference in how the isomers affect hMSC and 3T3-L1 cells under the differentiation process. However, neither isomer induces cell death in more mature cell types, such as differentiated 3T3-L1 adipocytes and H4IIE liver cells. Apoptosis and necrosis are induced by PGG, but the induction of apoptosis is the principle action of PGG.

As shown by our study, both isomers activate insulin receptors to suppress PEPCK transcription in H4IIE cells, but the PGG’s induction of pre-adipocyte cell death is independent of its insulin mimetic action. Therefore, our study is not in agreement with a previous study which found that PGG activated the insulin receptor, resulting in increased p53 and apoptosis in cancer cells [18]. The finding that PGG was unable to activate the insulin-growth factor receptor as insulin suggests that PGG and insulin might have different activities in promoting mitogenesis [17]. Activation of caspase-12 cleavage in PGG-treated pre-adipocytes shows that unfolded protein response (UPR) and endoplasmic reticulum stress may be involved in PGG-induced apoptosis [19,20]. Activation of caspase-6 also suggested the involvement of caspase-3 in PGG-induced cell death. This is demonstrated by studies showing that penta-1,2,3,4,6-O-galloyl-β-_D_-glucose induces a senescence-like growth arrest in human cancer cells by inducing autophagy and inhibits aggressive phenotypes of HepG2 cells by inducing G1 arrest and activating the caspase-3 cascade [21,22].

When an adipogenesis pathway is activated, PGG treatment promotes cell death and inhibits adipocyte TG accumulation in survival cells; thus, survival cells have a lower triglyceride content than control cells. This result is consistent with the data previously published [23]. However, although adipocyte morphology and triglyceride content were affected, biomarkers indicating cell fate are not completely altered in the present investigation. While pref-1 expression of survival cells was decreased after PGG treatment, PGG treatment actually increases aP2 protein expression in survival cells. aP2 is the protein first detected in adipose tissue and mature adipocytes [24]. Conversely, perilipin is a lipid droplet coating protein in adipocytes [16]. Therefore, reduced perilipin expression in PGG- (10 μM) treated cells correlates with the morphology in survival cells. As a result, the current data indicate that PGG treatment is unlikely to alter cell fate. Instead, PGG-mediated cytotoxic effects and alternation of proteins related to lipid metabolism favour the changes in morphology and cellular triglyceride content in survival cells [25]. PGG does not induce apoptosis nor a reduction in triglyceride content in fully differentiated adipocytes. However, it significantly lowers free fatty acid release from adipocytes. PGG can activate insulin receptors to facilitate insulin actions on adipocytes [11]. Therefore, this study suggests that PGG treatment reduces sub-sequential lipolysis and fatty acid release in differentiated 3T3-L1 adipocytes. Alleviation of fat accumulation in *Caenorhabditis elegans* by PGG has also been previously shown by Zhang et. al. and has been proposed as the anti-obesity mechanism for PGG [26]. Our current data in part support such a hypothesis.

The cell-based studies here show that PGG may prevent obesity in the early stages of the adipogenesis process and by attenuating lipid accumulation. The effective dosage of PGG that produces cytotoxicity and inhibits lipid accumulation in the early adipocyte lifecycle is in the 2.5–10 μM range. In contrast, activation of the insulin receptor by PGG to facilitate diabetes intervention requires higher doses up to 100 μM [11]. In order to correlate our current cell work to in vivo condition, the in vivo experimental design for this study uses lower doses of PGG (0.1 and 5 mg/kg) after mice have received high-fat feeding for two weeks. There is no statistical difference in the body weights of DIO mice that received PGG treatment and those that did not. However, energy intake was significantly reduced by PGG treatment. The reasons for this are not entirely clear. However, more detailed studies which examine the effects of PGG on fat/lean mass, nutrient uptake, feed conversion rate and gut microbiota composition would be needed to better understand why this is the case. Nevertheless, PGG treatment in DIO mice clearly improves a variety of obesity-related parameters, including dissected appearance, colon length, serum lipids, adipocyte size and lipid content, hepatic triglyceride accumulation and inflammation, insulin sensitivity, glucose tolerance, islet hypertrophy and insulin content and endocrine profile.

The beneficial outcomes for DIO mice treated with PGG are multifaceted and involve interactions between organs and systems. The cellular assays show that PGG treatment affects the early adipocyte lifecycle, and this study shows that there is a reduction in hypertrophic fat cells and adipose triglyceride content for DIO mice that are treated with PGG. The decreased release of fatty acid by PGG-treated 3T3-L1 adipocytes is also consistent with the reduced serum lipid levels observed in DIO mice treated with PGG. In obesity, an increment of fatty acids, glycerol, hormones, pro-inflammatory cytokines can promote peripheral insulin resistance, which leads to glucose intolerance, beta-cell dysfunction, and type 2 diabetes [27]. Inhibitory effects of PGG on early adipocyte lifecycle and lipid accumulation can attribute to the improvement in insulin sensitivity and glucose tolerance in vivo.

DIO mice that are treated with PGG also exhibit decreased energy intake, potentially contributing to its anti-obesity effects. However, a limitation of the current study is that pair feeding studies controlling energy intake were not undertaken. In the current study, reduced serum fatty acid levels and less hypertrophic adipocytes due to PGG treatment may reduce hepatic steatosis. The reduction of hepatic uncoupling protein 2 (*Ucp2*) gene expression may play a role, since it is thought to play a role in attenuating fatty liver disease in rats [28]. An increase in nitric oxide synthase 2 (*Nos2*) expression is also positively related to the development of liver fibrosis [29]. Overall, the reduction of hepatic *Ucp2* and *Nos2* expression in DIO mice treated with PGG indicated a preventive effect on obesity but also on non-alcoholic steatohepatitis.

High-fat feeding induces obesity, resulting in metabolic perturbation and dysregulation of endocrine hormones. Hypertrophy of pancreatic islets typically occurs in subjects that exhibit mild glucose intolerance, particularly when this occurs as a result of insulin resistance. This can subsequently eventually lead to beta-cell dysfunction and then the onset of type 2 diabetes. PGG treatment appears to inhibit this progression by attenuating insulin resistance and restoring islet morphology. Reduced energy intake may also play a role in maintaining homeostasis of endocrine hormones.

Shortening of the colon in DIO mice is thought to result from tissue inflammation, an impaired gut–brain axis, altered gut microbiota, and impaired GLP-1 release [30,31]. This study shows that a restoration of colon length is accompanied by an increase in serum GLP-1 levels in DIO mice that are treated with PGG. This is evidenced by the finding that the key GLP-1 degradation enzyme, DPP-4, is also inhibited by PGG in a dose-dependent manner, as shown in the Supplementary data (Figure S2). Restoration of GLP-1 levels would have several downstream effects, including the stimulation of insulin secretion (in a glucose-dependent manner), preservation of beta-cell function, expression and release of insulin-sensitizing adipokines, (e.g., adiponectin), and improved appetite control [32,33,34]. Therefore, restoration of gut health and an increase in incretin hormone levels due to PGG treatment may also play a role in maintaining glucose homeostasis in DIO mice. It is not yet known whether changes in gut microbiota due to a restoration of colon health play a role in incretin-mediated modulation of appetite via hypothalamus, but this is worthy of further investigation [35,36].

## 4. Materials and Methods

### 4.1. Preparation of 1,2,3,4,6-Pentagalloylglucose (PGG)

The biological activity between 1,2,3,4,6-Pentagalloylglucose (PGG) isomers was compared using chemically synthesized 1,2,3,4,6-Penta-O-galloyl-α-d-glucose (α-PGG) and 1,2,3,4,6-Penta-O-galloyl-β-d-glucose (β-PGG). The procedures were based on those of previous studies [37,38,39].

For in vivo studies, 1,2,3,4,6-Penta-O-galloyl-β-d-glucose was isolated from Paeoniae radix rubra, which was purchased from a local Chinese Medicine Pharmacy in Taipei city, Taiwan. This was identified as the roots of *Paeonia lactiflora* Pall. The ground material (150 g) was reflux-extracted twice using 0.6 L of 50% MeOH(aq) for 1 h each time. The supernatant was filtered through filter paper, combined and dried under the reduced pressure.

The crude extract (24.9 g) was obtained using an absorption-detaching process. Then, 70 g silica gel (40–63 μm; Merck, Darmstadt, Germany) was mixed with partially dried crude extract, and dried under reduced pressure. Dichloromethane, methanol, ethyl acetate, and 0.1% acetic acid were used as washing solutions. The E2 fraction was detached using ethyl acetate: methanol: 0.1% acetic acid = 15:2:0.5. The E2 fraction was further purified using a Sephadex LH-20 column with methanol as the mobile phase to isolate w6 (795.8 mg). w6 was obtained as a pale brown, amorphous powder and was identified as 1,2,3,4,6-Penta-O-galloyl-β-d-glucose (PGG) using nuclear magnetic resonance spectroscopy and mass spectrometry [12,40].

The chemical structure and purity of PGG isomers are shown in Figure S1A. The purity of 1,2,3,4,6-penta-O-galloyl-α-d-glucopyranoside was confirmed by NMR and Mass data. The α-anomer of C-1 glucose moiety was confirmed by the ^1^H-NMR chemical shift of δ 6.74 and its coupling constant of 3.64 Hz. (Figure S1B). The purity of 1,2,3,4,6-penta-O-galloyl-β-d-glucopyranoside, isolated from *Paeonia lactiflora*, is 98.17% by UPLC analysis at 210 nm. The identification was made by NMR and mass data. The β-anomer of C-1 glucose moiety was confirmed by the ^1^H-NMR chemical shift of δ 6.23 and its coupling constant of 8.3 Hz. (Figure S1C). The purity of α-PGG and β-PGG is 98.15% and 98.17%, respectively.

### 4.2. Cell Culture

H4IIE-C3 rat liver cell line (ATCC-CRL-1600) was cultured in Dulbecco’s Modified Eagle Medium (DMEM; 1 g glucose/L; Gibco, Waltham, MA, USA) containing 10%(*v*/*v*) foetal bovine serum (FBS; Gibco) and 1%(*v*/*v*) penicillin/streptomycin (P/S; Gibco). Human mesenchymal stem cells (hMSC) were cultured in DMEM (1 g glucose/L) containing 20%(*v*/*v*) FBS and 1%(*v*/*v*) P/S. Then, 3T3-L1 fibroblasts (ATCC-CL-173) were cultured in DMEM (4.5 g glucose/L) containing 10%(*v*/*v*) new bovine calf serum (CS; Gibco) and 1%(*v*/*v*) (P/S).

To induce 3T3-L1 adipogenesis, 3T3-L1 fibroblasts were seeded into 12-well plates at a density of 2 × 10^4^ cells/well. The culture medium was changed every two days until the 3T3L-1 preadipocytes reached confluency. An induction medium consisting of IBMX (0.5 mM; Sigma, St. Louis, MO, USA), insulin (0.5 mM; Novo Nordisk, NJ, USA) and dexamethasone (0.25 μM; Sigma) in DMEM with 10%(*v*/*v*) FBS and 1%(*v*/*v*) P/S was used for two days. Insulin (0.5 mM) in DMEM medium with 10%(*v*/*v*) FBS and 1%(*v*/*v*) P/S was then used for an additional two days, prior to culturing differentiated 3T3L-1 adipocytes in DMEM with 10%(*v*/*v*) FBS and 1%(*v*/*v*) P/S.

### 4.3. Viability Test

Cell viability after treatment was determined by MTT (3-(4,5-dimethylthiazol-2-yl)-2,5-diphenyltetrazolium bromide; Sigma) assay. The culture medium was replaced with a medium containing MTT and cultured for an additional 4 h at 37 °C. The converted insoluble formazan product was then dissolved in DMSO and the O.D. was measured at 570/630 nm by microplate reader.

### 4.4. Cell Cycle Analysis

At the end of the treatment, cells were trypsinized and resuspended in PBS for washing. The cell pellets were collected after centrifugation with 1000× *g* for 5 min and were further resuspended with 750 μL ethanol and maintained at −20 °C overnight. These cells were washed again with PBS and resuspended with 1 mL of 1%(*v*/*v*) Triton X-100. After 15 min, cell pellets were prepared and stained with 500 μL of Propidium Iodide (PI) staining solution, prior to flow cytometry analysis using a FACScan (Becton Dickinson, NJ, USA).

### 4.5. Annexin V and PI Staining

Cells were trypsinized and resuspended in culture medium at a concentration of 1 × 10^6^ cells/mL. Then, 500 μL of the cell suspension was transferred to a new tube, and 10 μL media binding reagent and 1.25 μL annexin V-FITC (Thermofisher scientific, Waltham, MA, USA) were added. The mixture was gently vortexed and incubated for 15 min at RT in the dark. After centrifuging at 1000× *g* for 5 min, the medium was removed. Next, 500 μL of cold 1× binding buffer and 10 μL PI solution were added. Following a gentle vortex, the samples were analysed on a FACScan within 1 h. The percentages of apoptotic and necrotic cells were calculated for each sample.

### 4.6. Oil-Red O Staining

At the end of the treatment, 3T3-L1 adipocytes were washed with PBS twice and fixed with 4%(*w*/*v*) paraformaldehyde for 1 h at 4 °C. After washing with PBS twice, cells were then stained with oil-red O solution (0.3%) for 30 min. After washing with PBS twice, the morphology of stained cells was determined using a microscope. Next, 4% Igepal CA-630 (Sigma) in isopropanol solution was used to extract triglyceride. The oil content was quantified at a wavelength of 520 nm using microplate reader.

### 4.7. Western Blot

The cell lysates were prepared by using ice-cold lysis buffer (25 mM Tris/HCl (pH 7.4), 50 mM NaF, 100 mM NaCl, 1 mM sodium vanadate, 5 mM EGTA, 1 mM EDTA, 1% (*v*/*v*) Triton X-100, 10 mM sodium pyrophosphate, 1 mM benzamidine, 0.1 mM PMSF, 0.27 M sucrose, 2 μM microcystin, and 0.1% (*v*/*v*) 2-mercaptoethanol) containing a protease inhibitor cocktail. Lysates were collected from the supernatant part after a 10 min centrifugation at 13,000× *g*. The cell lysates were separated using 10% SDS-PAGE and transferred to an activated polyvinylidene difluoride (PVDF) membrane (Merck Millipore, Burlington, MA, USA). The membrane was blocked with 5% non-fat milk in TBST and was incubated overnight at 4 °C with TBST that contained the required primary antibody. After replacement with the corresponding secondary antibody, an enhanced chemiluminescence (ECL) kit (Amersham Biosciences, Piscataway, NJ, USA) was used. The primary anti-bodies were: Caspase 12 total and cleaved form (Cell Signaling Technology, Beverly, MA, USA, #2202), Caspase 6 (Cell Signaling Technology, #9762), Caspase 6-cleaved (Cell Signaling Technology, #9761), PARP-cleaved (Cell Signaling Technology, #9544), Pref-1 (SantaCruz biotechnology Inc., Heidelberg, Germany, #25437), Perilipin (SantaCruz biotechnology, #67164), Actin (Chemicon, Temecula, CA, USA, #MAB1501), and FABP4/aP2 (R&D systems, #AF1443). The image of the Western blot result was captured by the chemiluminescent imaging system, KETA C series, equipped with chemiluminescent image capture and analysis software, Magic Chemi (WALTEC, Taipei, Taiwan).

### 4.8. Animal Studies

Male C57BL/6 mice were purchased from the National Laboratory Animal Center, Taipei, Taiwan. Animals were handled in accordance with the *Guide for the Care and Use of Laboratory Animals* (Institute of Laboratory Animal Resource, 1996), and the protocol for animal experiments was approved by the Institutional Animal Care and Management Committee of the National Research Institute of Chinese Medicine, Taipei, Taiwan (IACUC N: 108-370-1). Animals were housed in a regulated vivarium (22 °C ± 23 °C) at 46–60% relative humidity with 12 h light and dark cycles and free access to water and food.

For the experiment, eight-week-old male C57/BL6 mice were grouped (*n* = 6) as: Group 1: Controlled diet (NCD); Group 2: High-Fat Diet (HFD); Group 3: HFD + PGG (0.1 mg/kg); Group 4: HFD + PGG (5 mg/kg). The diet was manipulated for two weeks, and then PGG was oral gavage for 12 weeks. During these 12 weeks, the weekly body weight, food intake and fasting (for 16–18 h) blood glucose were measured.

### 4.9. Pyruvate Tolerance Test

Mice were fasted overnight for 16–18 h and an i.p. injection of a sodium pyruvate solution (2 g/kg body weight) was administered. Blood glucose was measured 0, 15, 30, 60 and 120 min thereafter. The area under curve (AUC) was determined from individual blood glucose vs. time curve by employing GraphPad Prism 8.0 (GraphPad, San Diego, CA, USA).

### 4.10. Glucose Tolerance Test

Mice were fasted overnight for 16–18 h, and a glucose solution (3 g/kg body weight) was administrated orally. Blood glucose was measured 0, 15, 30, 60 and 120 min thereafter. Individual AUC was determined using method described above.

### 4.11. Insulin Tolerance Test

Mice were fasted for 6 h, and an i.p. injection of an insulin solution (0.25 U/kg body weight) was given. Blood glucose was measured 0, 15, 30, 60 and 120 min thereafter. Individual AUC was determined using the method described above. The individual area above curve was obtained by subtracting individual AUC from the maximum AUC data from HFD group.

### 4.12. Biochemical Parameters

Free fatty acids and triglyceride were determined using commercial assay kits (BioVision Inc. Milpitas, CA, USA). Blood glucose was measured at the tail tip using an electrode-type glucometer (Horiba Inc., Kyoto, Japan). Bio-Plex Pro™ mouse diabetes immunoassays (Bio-Rad Laboratories AB, Hercules, CA, USA) were used for endocrine profiling for fasting serum samples, including insulin, GLP-1_active_, ghrelin, leptin, and adiponectin (*n* = 4 per group, duplicate samples, randomly selected from each group to fit the assay plate). The results were calculated using the software provided by the vendor (Bio-Rad). A homeostatic model was used to assess β cell function (HOMA-B%) = 20 × fasting insulin (µU/mL)/fasting blood glucose (mM) − 3.5.

### 4.13. Histological Examinations

For the histological examination, pancreatic tissues were fixed in 4% paraformaldehyde, and 3 μm paraffin sections were stained with haematoxylin and eosin (H&E). To determine islet insulin content, paraffin sections were stained with primary antibodies (1:200) against insulin (Merck Millipore, KGaA, Darmstadt, Germany). After washing, a secondary antibody conjugated to horseradish peroxidase (Dako EnVision K4065, Glostrup, Denmark) was used. The result was visualized using the DAB reagent (Dako EnVision) in the presence of Mayer’s haematoxylin staining. Images were captured using a Nikon E600 microscope that is equipped with a digital camera (SAGE vision SG-5.07, Nikon, Tokyo, Japan) [41].

### 4.14. Real-Time PCR

Mice were sacrificed, and liver samples were lysed with the TRI-reagent to extract total RNA. Then, 1 μg of total RNA was reverse-transcribed to generate templates. Afterward, 25 ng of cDNA was mixed with primer sets and the LightCycler^®^ 480 SYBR Green I Master mix (Roche Life Science, Indianapolis, IN, USA) and placed in a LightCycler^®^ 480 Multiwell plate. PCR was performed using the LightCycler 480 system with annealing conditions of 60 °C for 10 s. The primer sets were:

b-actin: forward 5′-CTAGAAGCACTTGCGGTGCAC-3′ and reverse 5′-GAAATCGTGCGTGACATCAAA-3′; FASN: forward 5′-CACAGTGCTCAAAGGACATGCC-3′ and reverse 5′-CACCAGGTGTAGTGCCTTCCTC-3′; CPT-1: forward 5′-GGCATAAACGCAGAGCATTCCTG-3′ and reverse 5′-CAGTGTCCATCCTCTGAGTAGC-3′; UCP2: forward 5′-TAAAGGTCCGCTTCCAGGCTC-3′ and reverse 5′-ACGGGCAACATTGGGAGAAGTC-3′; NOS-2: forward 5′-GAGACAGGGAAGTCTGAAGCAC-3′ and reverse 5′-CCAGCAGTAGTTGCTCCTCTTC-3′; IL-1β: forward 5′-TGGACCTTCCAGGATGAGGACA-3′ and reverse 5′-GTTCATCTCGGAGCCTGTAGTG-3′; IL-6: forward 5′-TACCACTTCACAAGTCGGAGGC-3′ and reverse 5′-CTGCAAGTGCATCATCGTTGTTC-3′.

### 4.15. Statistics

Data were presented as means ± standard error of the mean. Statistical analysis was performed using GraphPad Prism 8.0 (GraphPad). Single parameter-based comparisons used unpaired Student’s *t* tests. Multiple group comparison used ordinary one-way ANOVA with Dunnett’s multiple comparisons test. NCD group and HFD (none) were used as a control group to execute multiple comparisons. *p* value of less than 0.05 was considered to be significant.

## 5. Conclusions

In conclusion, PGG reportedly occurs in a wide range of plants and herbs. Oral acute toxicity dose of PGG is estimated to be greater than 500 mg/kg in rats [9,42]. Therefore, oral dosage of PGG at the levels used in the current study are considered to be safe for humans, and therefore it is feasible for future clinical applications or development into a dietary supplement. This study has demonstrated that repeated PGG treatment interrupts the adipocyte lifecycle in the early stages of adipogenesis by promoting apoptosis and necrosis, an action which appears to be independent of insulin signalling. There was no significant weight loss, but fat cell hypertrophy, hyperlipidaemia, steatohepatitis and glucose intolerance in DIO mice were significantly attenuated by β-PGG administration in vivo (5 mg/kg). Therefore, the consumption of PGG could be beneficial in the clinic for treating and managing obesity at its earliest stages.

## Data Availability

All data are reported in the manuscript.

## Supplementary Materials

The following supporting information can be downloaded at: https://www.mdpi.com/article/10.3390/ijms23074052/s1.
