# Supplementary material for: 1,2,3,4,6-Penta-O-galloyl-d-glucose Interrupts the Early Adipocyte Lifecycle and Attenuates Adiposity and Hepatic Steatosis in Mice with Diet-Induced Obesity
Source: Int J Mol Sci. 2022 Apr 6;23(7):4052. doi: 10.3390/ijms23074052 (PMC8999501; doi:10.3390/ijms23074052)
Supplement: Supplementary file 1 [file ijms-23-04052-s001.zip › ijms-1576015-supplementary.pdf]

Supplementary Materials:

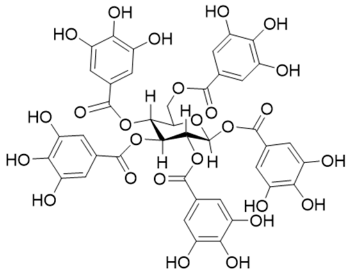

1,2,3,4,6-penta-*O*-galloyl-β-D-glucopyranoside

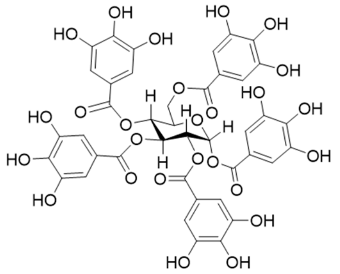

1,2,3,4,6-penta-*O*-galloyl-α-D-glucopyranoside

(A)

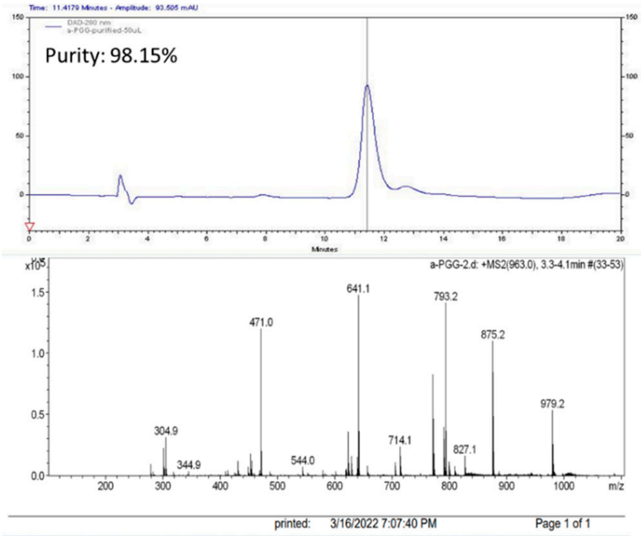

1,2,3,4,6-penta-O-galloylα-D-glucopyranose, 1H NMR (400 MHz, Acetone-d6)

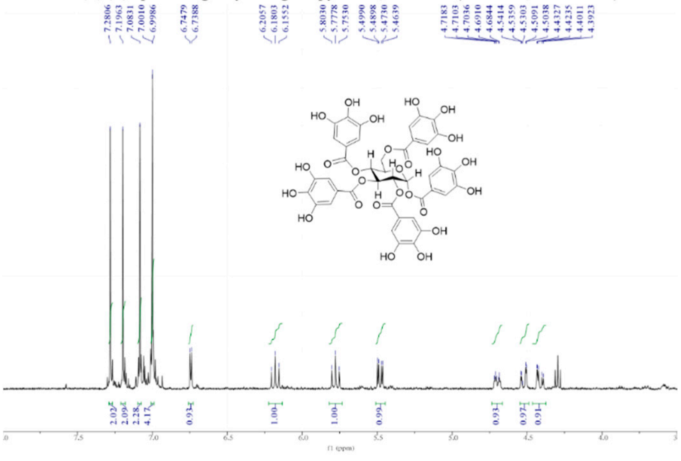

(B)

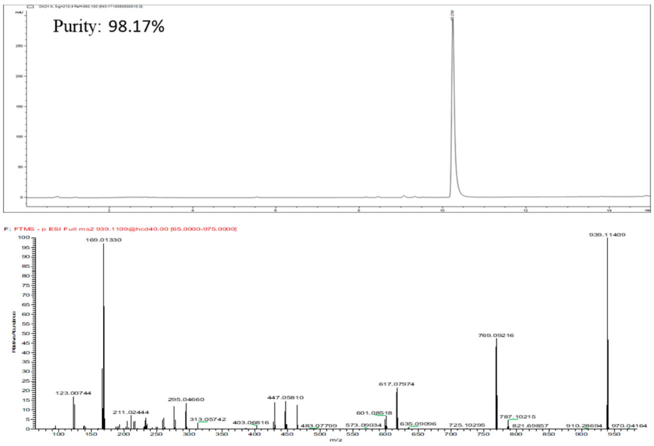

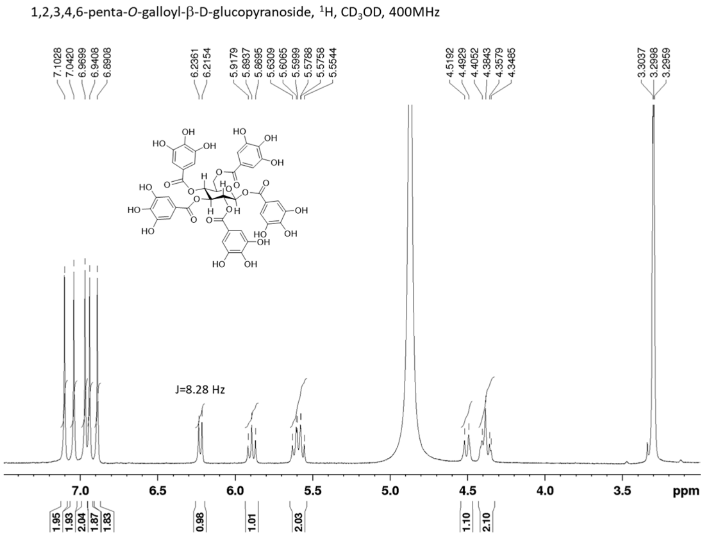

(C)

Figure S1. Characterization of PGG isomers used in the study. (A) Chemical structures of of α-PGG and β-PGG. (B) The purity of 1,2,3,4,6-penta-O-galloyl-α-D-glucopyranoside was determined by Mass and NMR. The analyses were performed by HPLC and C-18 column was used. The flow rate is 1mL/min. The mobile phase for the purification of pentagalloylglucose (PGG) consisted of acetonitrile and 0.1% (v/v) formic acid at a ratio of 20:80 (v/v). The absorption wavelength was 280nm. (C) The purity of 1,2,3,4,6-penta-O-galloyl-β-D-glucopyranoside was determined by Mass and NMR. The analyses were performed on an Agilent 1260 UPLC system including Binary Pump, Multisampler, Thermostat and Diode Array Detector. The chromatographic data were collected and analyzed by Agilent OpenLAB CDS software. The Thermo Syncronis C18 column (100 × 2.1 mm, 1.7 μm) was used. The column and sample temperature were maintained at 40 and 25 °C, respectively. The mobile phase consisted of water (0.1% phosphate buffer) (A) and acetonitrile (B) at a flow rate of 0.4 mL/min. The linear gradient was applied as following: 2% A, hold for 1 min; 2%–10% A, 1–2 min and hold for 2 min; 10%–25% A, 4–10 min; 25%–50% A, 10–14 min. The separation was followed by a 3-min washing procedure. The injection volume was 1 μL. The detection wavelength was set between 200 and 400 nm, and the chromatographs were extracted at 210 nm for purity check. 1,2,3,4,6-O-Penta-O-galloyl-β-D-glucopyranoside (PGG): a pale brown, amorphous powder; UV λmax at 211, 231 and 278 nm; (c) ESI-MS (negative ion mode) m/z 939.1141 [M − H]-.

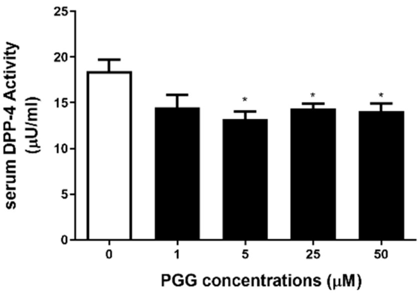

Figure S2. Effect of PGG on serum DPP-4 activity: Data are shown as mean±SEM (n=3). *P<0.05 compared with control (vehicle). The inhibitory effect of β-PGG on DPP-4 activity was analyzed using a fluorometric assay kit (BioVision, Milpitas, CA, USA), according to manufacturer's instructions. Mouse serum was prepared as the DPP-4 enzyme source, and activity was determined after a 30-min incubation at 37°C in the presence of vehicle or β-PGG. Result shows that PGG inhibits about 12% of serum DPP-4 activity at the concentration from 5 to 50μM.
